# Biosecurity implementation in poultry farms across Europe and neighboring countries: a systematic review

**DOI:** 10.3389/fvets.2025.1653543

**Published:** 2025-09-19

**Authors:** Ronald Vougat Ngom, Marta Leite, Giuditta Tilli, Andrea Laconi, Qamer Mahmood, Jasna Prodanov-Radulović, Alberto Allepuz, Ilias Chantziaras, Alessandra Piccirillo

**Affiliations:** ^1^Department of Animal Production, School of Veterinary Medicine and Sciences, University of Ngaoundere, Ngaoundere, Cameroon; ^2^Associated Laboratory for Green Chemistry (LAQV) of the Network of Chemistry and Technology (REQUIMTE), Faculty of Pharmacy, University of Porto, Porto, Portugal; ^3^Department of Comparative Biomedicine and Food Science, University of Padua, Viale Legnaro, Italy; ^4^Department of Comparative Biomedicine and Food Science, University of Padua, Viale Legnaro, Italy; ^5^Veterinary Epidemiology Unit, Department of Internal Medicine, Reproduction and Population Medicine, Faculty of Veterinary Medicine, Ghent University, Merelbeke, Belgium; ^6^Scientific Veterinary Institute Novi Sad, Novi Sad, Serbia; ^7^Departament de Sanitat i Anatomia Animals, Universitat Autònoma de Barcelona (UAB), Bellaterra, Barcelona, Spain

**Keywords:** biosecurity, assessment, prevention, poultry, Europe, Israel, Tunisia, Turkey

## Abstract

**Introduction:**

Modern poultry production systems inherently concentrate large numbers of birds, which also increases the risk and potential impact of disease outbreaks. Biosecurity is widely recognized as the most important tool for reducing the risk of disease introduction, establishment, and spread to, within, and from an animal population. Thus, effective biosecurity is essential for sustainable poultry production, and assessing its implementation represents a crucial step. This systematic review aimed to evaluate biosecurity implementation in poultry farms across European and neighboring countries

**Methods:**

The Cochrane Handbook and PRISMA 2020 guidelines were followed to perform the systematic review.

**Results:**

Of the 1,515 articles retrieved from four databases, only 44 met the inclusion criteria and 16 provided usable data for assessing biosecurity implementation. Despite relatively broad geographical coverage, including eight multi-country studies involving 36 national assessments, the distribution of studies was uneven. Moreover, most studies (77%) were pathogen- or disease-specific (e.g., *Campylobacter* spp., avian influenza, etc.) and focused on a single poultry species, primarily broilers (55%), while assessments involving minor poultry species were rare. There was also marked variability in the methods used to assess biosecurity, and the level of biosecurity implementation differed significantly across countries. Based on descriptive evaluations, 58% of farms implemented all the biosecurity measures assessed. According to scoring-based assessments, the overall average biosecurity score was 66.9 out of 100. The most frequently implemented measures were those related to infrastructure and control of biological vectors, disease management, and purchase of one-day-old chicks.

**Discussion:**

The heterogeneity of results, driven by differences in study design, poultry species, production systems, and methodological approach, highlights the complexity of evaluating biosecurity across diverse national contexts. This variability may reflect differences in epidemiological conditions, research funding, and national priorities. Although this review focused solely on primary research studies, the findings underscore the need to promote cross-country collaboration to enhance knowledge sharing and data harmonization.

## 1 Introduction

Among the major contributors to global poultry meat and egg production, European (EU) countries play a significant role in setting high standards for quality and animal welfare, serving as both a major producer and supplier to international markets (1). Achieving high levels of production is the result of a combination of production policies, market regulations, integrated health and production management strategies. Biosecurity is widely recognized as a key tool for preventing the introduction of infectious diseases (external biosecurity) and the establishment and spread (internal biosecurity) from and within an animal production site, which in turn reduces antimicrobial usage and enhances animal production performance (2–4). Although all EU member states are subject to the same overarching legal framework for animal health (5), the practical implementation of biosecurity measures can vary significantly across countries (2, 6–8). These differences reflect the diverse needs of national poultry sectors and are expressed through tailor-made national biosecurity legislation (9) and/or country-specific quality label regulations (10, 11). The implementation of biosecurity measures also depends on various country-specific factors, such as geographical location of farms, their structural characteristics (e.g., size, production type, and category), the national epidemiological situation (e.g., presence, prevalence, or risk profile of poultry diseases), available financial resources, and the awareness and knowledge of stakeholders regarding biosecurity (12–14). A further challenge is the scarcity of available data on the actual level of biosecurity implementation in poultry farms across European countries (15–17). This data gap represents a bottleneck for the design of effective interventions aimed at improving biosecurity implementation (9, 12, 18). In response to these knowledge gaps, the COST Action CA20103 - Biosecurity Enhanced Through Training Evaluation and Raising Awareness (BETTER) - was launched in 2021. One of its aims is to gather and consolidate knowledge on biosecurity regulatory frameworks.^1^ To this end, an overview of biosecurity implementation as mandated by national legislation and other regulatory frameworks in intensive poultry production was performed (8). The analysis revealed a general lack of data on the actual level of implementation of biosecurity measures in most countries. Therefore, a comprehensive and systematic assessment was needed to address this important gap. The objective of this study was to assess the level of biosecurity implementation in poultry farms across key European countries contributing to the global poultry market. In addition to European COST member countries, Turkey (Full COST Member), Tunisia (Near Neighbor Country), and Israel (Cooperating Member), were also included in the analysis.

## 2 Methods

This systematic review was conducted in accordance with the Cochrane Handbook for Systematic Reviews (19) and is reported in line with the Preferred Reporting Items for Systematic Reviews and Meta-Analyses (PRISMA) guidelines (20).

### 2.1 Protocol and registration

The protocol was developed according to PRISMA-P guidelines (21) and was archived in the University of Padua’s Research Archive institutional repository.^2^ It was also registered on the Systematic Reviews for Animals and Food (SYREAF) website.^3^

### 2.2 Information sources and search strategy

Four databases were searched: Web of Science (WOS) and PubMed via the University of Padua in Italy, and Agricola (Proquest) and CAB Abstract (Ovid) via the University of Bern in Switzerland. No publication date restrictions were applied. The search targeted primary research assessing biosecurity implementation in poultry farms across European and neighboring countries. The strategy followed the PICO framework, and employed a multi-stranded approach, using the following search terms: [Poultry] AND [Biosecurity] AND [Implementation] AND [European Countries OR Turkey OR Tunisia OR Israel]. The detailed search strategy, carried out in CAB Abstract on December 20, 2023, is reported in Supplementary material 1.

Studies published in English, Spanish, or French and reporting primary data on biosecurity implementation in intensive poultry farms (broiler, layer, duck, goose and turkey) within the selected countries were considered. In line with a protocol deviation, randomized controlled trials, cohort studies, and case–control studies were excluded. Studies involving experimentally challenged animals or model-based designs were also excluded.

### 2.3 Study selection

All identified records were deduplicated using Zotero (version 7.0), and screened using Rayyan.^4^ Six reviewers, working in pairs, independently screened titles and abstracts. Full texts of potentially eligible studies were retrieved and assessed. Each pair screened one-third of the articles, and calibration exercises were conducted prior to each step using randomly selected papers. Discrepancies between pairs of reviewers were resolved through discussion or with a third reviewer.

Eligibility was determined using the following criteria:

1. Is the publication in English, French, or Spanish? Yes [Include], No [Exclude]
2. Is the full text available? Yes [Include], No [Exclude]
3. Is the article original research? Yes [Include], No [Exclude], Unclear [Include]
4. Does it concern broilers, layers, turkeys, breeders, ducks, or geese? Yes [Include], No [Exclude]
5. Does it concern intensive poultry farming? Yes [Include], No [Exclude]
6. Does it assess biosecurity implementation at farm level? Yes [Include], No [Exclude]
7. Is the study conducted in Europe, Israel, Tunisia, or Turkey? Yes [Include], No [Exclude]
8. Is the publication a randomized controlled trial, case–control, or case-series study? No [Include], Yes [Exclude]

### 2.4 Data extraction

Data extraction was performed by six reviewers working in pairs, each handling one-third of the included studies. A Microsoft Excel^Ⓡ^ Spreadsheet (version 2020), developed by two authors and validated during a calibration phase on five randomly selected papers, was used. Extraction was done independently, and conflicts were solved as previously described. As a deviation from the protocol, only studies with data collection occurring from 2010 onward were included for biosecurity-related data, to ensure consistency with current legislative frameworks. Studies using models to estimate biosecurity levels were excluded, based on the assumption that non-model-based studies better reflect field conditions.

### 2.5 Data items

The following information was extracted from each study: year of publication; country; study time-frame; poultry category (broilers, layers, ducks, turkeys, breeders, other minor species); number of farms and number of flocks; production type (e.g., conventional, organic, antibiotic-free, outdoor, free-range, multi-species); type of analysis (e.g., scoring system, descriptive statistics, probability estimates using risk models or artificial intelligence); specific pathogen or disease (if relevant to biosecurity assessment). The full data extraction sheet is available upon request from the corresponding author.

### 2.6 Quality appraisal

Each included study was critically appraised using the tool developed by Downes et al. (22). This tool covers multiple aspects including study objective, methodology, results, and discussion. Appraisal was conducted independently by two reviewers, with discrepancies resolved through discussion.

### 2.7 Data synthesis

The selection process was summarized in the PRISMA flowchart. Descriptive statistics were used to summarize study characteristics. As described previously (23) and to avoid misclassification, for each study, biosecurity measures were grouped according to Biocheck. UGent™ poultry subcategories (purchase of one-day chicks, depopulation of broilers, feed and water, removal of manure and carcasses, visitors and farmworkers, material supply, infrastructure and biological vectors, location of the farm, disease management, cleaning and disinfection and materials and measurements between compartments) to ensure consistency across studies (24). For example, the subcategory “purchase of one-day-old chicks” included measures such as introduction of new animals, flock registration (origin, number of poultry), transport, number and health status of source herds; the “depopulation of broilers” category included measures like “all-in/all-out” poultry production on site and thinning. Studies were grouped based on the method used to assess biosecurity: (1) descriptive methods, where pooled results were expressed as the percentage of farms implementing specific categories of biosecurity measures; and (2) scoring methods, where pooled results were expressed as scores of biosecurity implementation. As described in the protocol, the intention of this review was to conduct a meta-analysis. However, due to the limited number of studies in each group and their heterogeneity, a meta-analysis, and therefore sensitivity analysis and publication bias assessment, were not conducted. For each biosecurity subcategory, the mean and interquartile range (IQR) were calculated.

## 3 Results

### 3.1 Study selection

**Figure 1:**
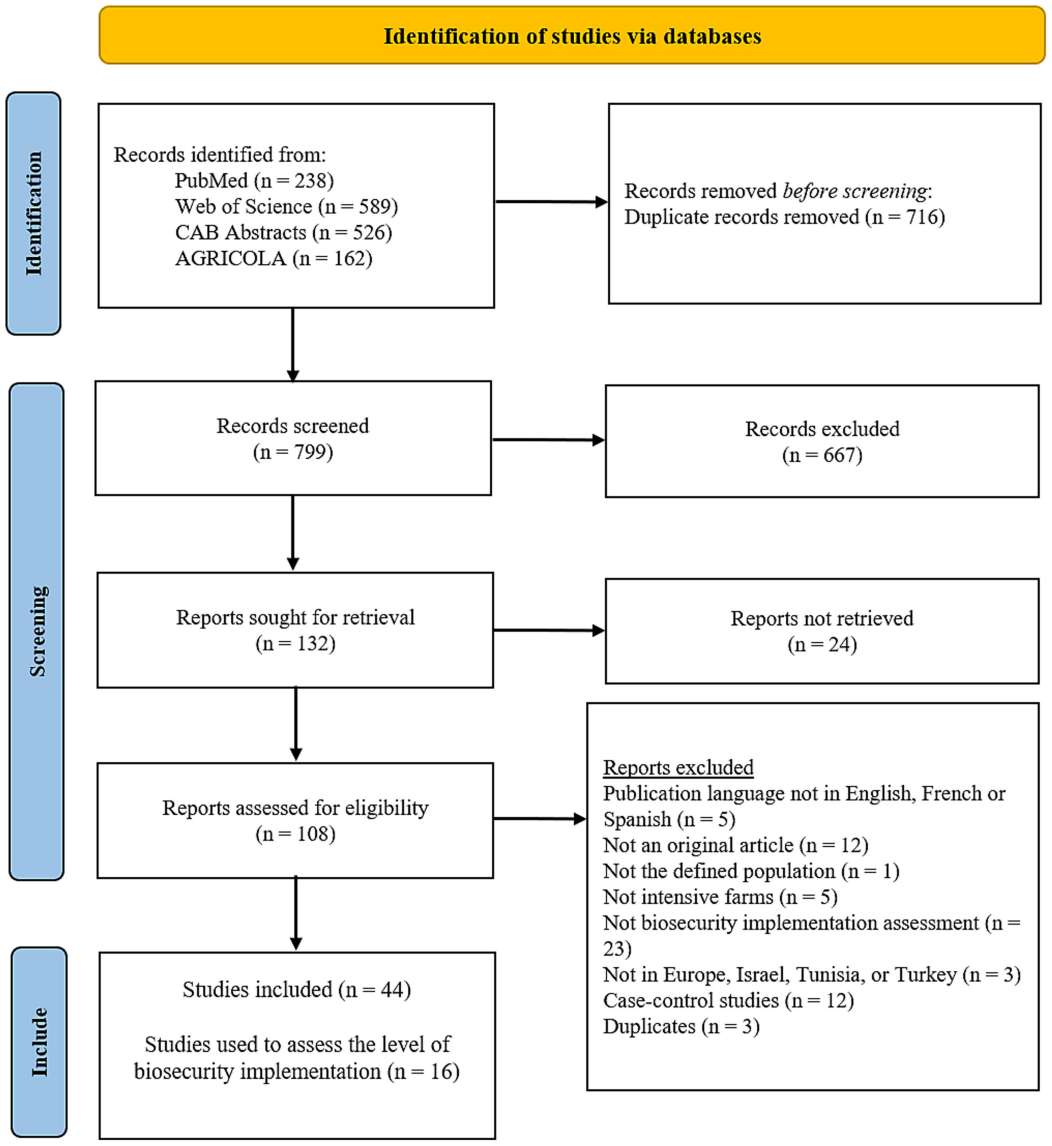
PRISMA flow diagram illustrating the selection process of studies included in the systematic review, from initial identification through screening and final inclusion. The process follows the PRISMA (Preferred Reporting Items for Systematic Reviews and Meta-Analyses) 2020 guidelines.

A total of 1,515 articles were retrieved from four databases. After removing duplicates, 799 unique records were screened. Following the selection process, 44 articles were included, of which only 16 contained extractable data on biosecurity implementation. During the full text screening, the majority of articles (23 out of 64) were excluded because they did not concern the assessment of biosecurity implementation. The flow of study selection is summarized in Figure 1.

### 3.2 Study characteristics

The characteristics of the included studies are reported in Table 1. Overall, there was an increasing trend in publications between 2000 and 2023. The study periods ranged from 1 to 43 months, with an average of 13 months. Among the countries represented, the United Kingdom had the highest number of studies (*n* = 10), followed by France and The Netherlands (*n* = 9 each), Denmark (*n* = 7), and Spain (*n* = 6). About 20% of countries were represented by only one study, including Cyprus, Finland, Greece, Ireland, Kosovo, Serbia, Sweden, Tunisia, and Turkey. In terms of geographical coverage, three studies focused on individual sites, 16 covered specific regions and nine provided national-level assessments. Eight studies were multi-country in scope, resulting in a total of 36 country-level analyses. Most studies focused on a single poultry species (77%), predominantly broilers (55%). The number of farms included in the studies ranged from 5 to 1,004 farms, with an average of 123. Biosecurity assessments for layers and turkeys appeared in 9% of studies, while 23% included more than one poultry category. Seventy-three percent of studies concerned conventional production systems. Only 10 out of 44 studies had biosecurity evaluation as their primary objective; the remainder (*n* = 6) focused on specific pathogens or diseases. The most frequently investigated topics included *Campylobacter* spp. (*n* = 12), antimicrobial resistance (*n* = 8), avian influenza (*n* = 8), *Escherichia coli* (*n* = 4), and *Salmonella* spp. (*n* = 2). Regarding methodological approaches to biosecurity assessment, 22 studies used descriptive analysis (e.g., percentage of farms implementing/having certain measures), nine applied scoring systems, and eight relied on probabilistic models. Five studies did not report any method for biosecurity assessment.

**Table 1:** Summary of key characteristics of the studies included in the systematic review.

Country	Study time-frame	Geographic coverage	Poultry category	Number of farms	Production type	Method of biosecurity evaluation	Pathogen/disease	References
Denmark	Dec 1995	NR	Broilers	55	NR	Descriptive	*Campylobacter* spp.	(42)
Denmark	Jun 2000 - Aug 2001	Local	Broilers	NR	Mixed	Descriptive	Haemolytic *Gallibacterium* spp.	(43)
France	March–July 2001	Regional	Turkeys	131	Conventional	Descriptive	AMR	(44)
Spain	Oct - Nov 2003	Regional	Broilers	33	NR	Descriptive	/	(45)
UK	Jun - Oct 2005 Feb - Nov 2006	National	Broilers	51	Conventional	Descriptive	*Campylobacter* spp.	(46)
UK	NR	NA	Broilers	150	NR	Scoring	*Campylobacter* spp.	(47)*
UK	Oct 2008	Local	More than one category	22	Mixed	Descriptive	Highly Pathogenic Avian influenza	(38)
Turkey	2005–2006	Local	Turkeys	71	Conventional	Descriptive	Highly Pathogenic Avian influenza	(48)
Finland	Feb to Dec 2007	NR	Broilers	22	Conventional	NR	/	(49)
France	Feb 2008 - April 2009	NR	Ducks	NR	Conventional	Probabilistic model	Avian influenza	(50)
UK	Oct 2006 - Sep 2007	National	Turkeys	329	Conventional	Probabilistic model	Resistant *Escherichia coli*	(51)
Netherlands	May - Dec 2009	NR	More than one category	42	NR	Scoring	Avian influenza	(40)
Tunisia	Oct 2010 - May 2011	National	More than one category	800	NR	Probabilistic model	Low Pathogenic Avian influenza	(52)
France	Jan - Dec 2008	Regional	Broilers	121	Conventional	Descriptive	*Campylobacter* spp.	(53)*
Belgium	May 2012 - Jan 2013	National	Broilers	13	Conventional	Scoring	/	(34)*
UK	Aug 2010 - Sep 2011	NR	Layers	60	Mixed	Descriptive	*Salmonella*	(54)*
Denmark, Norway	Dec 2010 - Jun 2011	Multi-country	Broilers	277	Conventional	Descriptive	*Campylobacter* spp.	(35)
Norway	Nov 2013 - Jan 2015	Regional	Broilers	27	Conventional	Probabilistic model	Cephalosporin-resistant *Escherichia coli*	(55)
Ireland	Jan 2013 - Jan 2014	Regional	Broilers	23	Conventional	Probabilistic model	*Campylobacter jejuni*	(56)
UK (England and Wales)	Jun 2001 - Jun 2003	Regional	More than one category	89	Conventional	Probabilistic model	*E. coli* and thermophilic *Campylobacter* spp.	(57)
Denmark, Netherlands, Norway, Poland, Spain, UK	NR	National	Broilers	NR	Conventional	Probabilistic model	*Campylobacter* spp.	(58)
Kosovo	May 2012 - Oct 2013	Regional	Layers	22	Conventional	Descriptive	*Salmonella* and *Dermanyssus gallinae*	(59)*
UK (England)	NR	Regional	Broilers	7	Conventional	Probabilistic model	/	(60)
Denmark	Jan 2012 - Dec 2013	National	Broilers	171	Conventional	Descriptive	*Campylobacter jejuni*	(61)
Netherlands	NR	NR	More than one category	5	Conventional	Probabilistic model	Highly Pathogenic Avian influenza	(29)
France	Feb - Jul 2016	Regional	Ducks	46	Conventional	Descriptive	/	(62)*
Belgium, Bulgaria, Danmark, France, Germany, Italy, Netherlands, Poland, Spain	May 2014 - Jun 2016	Multi-country	Broilers	176	Conventional	Scoring	AMR	(63)*
France	3 Mar - 6 Jun 2018	Regional	Ducks	127	Outdoor/Conventional	Scoring	Highly Pathogenic Avian influenza	(12)*
Spain	Autumn 2014 and Spring 2015	Regional	Broilers	14	Conventional	Descriptive	*Campylobacter* spp.	(30)*
Belgium, Netherlands	Sep 2017 - Apr 2018	Multi-country	Broilers	28	Conventional	Scoring	AMR	(64)*
Belgium, Netherlands	Sep 2017 - May 2019	Multi-country	Broilers	30	Conventional	Scoring	/	(65)
UK	May 2016 Jun 2017 - Jan 2018	Regional	Broilers	16	Conventional	NR	*Campylobacter*	(66)
UK (Scotland)	Mar - May 2017	National	More than one category	76	Mixed	Descriptive	/	(67)*
France	Oct 2016 - Sep 2018	National	Ducks	1,004	Conventional	Descriptive	/	(12)*
Germany, France, Spain	Oct 2014 - Oct 2016	Multi-country	Turkeys	60	Conventional	Descriptive	AMR	(36)*
Germany	April - Nov 2018	Regional	Broilers	100	Conventional	Descriptive	Cellulitis (*Escherichia coli*)	(68)
Sweden	1 Oct 2020–30 Sep 2021	National	More than one category	24	Conventional	NR	Highly Pathogenic Avian influenza	(39)
Netherlands	NR	NR	Broilers	NR	Conventional	Probabilistic model	*Campylobacter* spp.	(69)
Belgium, Bulgaria, Denmark, France, Germany, Italy, Netherlands, Poland and Spain	May 2014 - Jun 2016	Multi-country	Broilers	174	Conventional	Scoring	AMR	(70)
Italy	May 2018 - Jan 2021	Regional	More than one category	114	Mixed	Descriptive	*Chlamydia gallinacea*	(71)
Italy	2018–2019	Regional	More than one category	259	Conventional	Descriptive	/	(9)*
Serbia	Jun 2018 - Dec 2021	Regional	Broilers	100	Conventional	NR	*Eimeria* spp.	(72)
Italy	Apr - Sep 2021	National	More than one category	30	Conventional and free-range	Descriptive	/	(13)*
Netherlands, Greece and Cyprus	Dec 2019 - Mar 2021	Multi-country	Broilers	35	Conventional	Scoring	AMR	(73)*

/: papers focused exclusively on the identification of biosecurity measures; *: papers from which data were extracted to assess the level of biosecurity implementation; NR, not reported; NA, not available; AMR, antimicrobial resistance.

### 3.3 Results of individual studies

Out of the 44 articles included in the review, data extraction concerning biosecurity implementation was possible for only 16. The remaining 28 were excluded from synthesis due to the following reasons: five studies were published before 2010; 10 used probabilistic models; and 13 did not contain extractable data. As a result, all downstream analyses were performed on 16 papers. A summary of the extracted results is presented in Tables 2, 3.

**Table 2:** Results of the included studies using scoring systems to evaluate biosecurity implementation.

Variable	Fraser et al. (47)^#^	Gelaude et al. (34)	Luiken et al. (63)	Delpont et al. (12)^#^	Caekebeke et al. (64)	Schreuder et al. (73)^#^
*Total external biosecurity*	2.2	66.5	75^*^	61^*^	65.7	49.9
Purchase one-day-old chicks	/	69.0	/	/	67.9	43.0
Depopulation broilers	1.8	61.5	/	65.0	61.6	8.0
Feed and water	/	46.0	/	/	50.1	55.5
Removal manure and carcasses	/	66.5	/	80.0	69.7	63.8
Visitors and farmworkers	2.7	78.0	/	75.25	71.8	61.0
Material supply	/	45.0	/	/	52.1	/
Infrastructure and biological vectors	/	82.5	/	55.0	83.3	67.1
Location farm	/	74.5	/	79.0	58.4	50.7
*Total internal biosecurity*	2.6	75.0	59^*^	72.0^*^	59.9	/
Disease management	/	82.5	/	72.0	71.5	42.00
Cleaning and disinfection	2.8	69.0	/	65.0	55.1	76.3
Materials and measures between compartments	2.3	76.0	/	61.5	50.9	52.6
Total overall biosecurity	2.4	70.8	67.0	63.0^*^	63.9	57.0

#: A method other than the Biocheck. UGent™ assessment tool was used to evaluate biosecurity. *: Value directly extracted from the original paper. /: Data not available.

**Table 3:** Results of the included studies using descriptive methods to evaluate biosecurity implementation.

Variable	Allain et al. (53)	Gosling et al. (54)	Sylejmani et al. (59)	Delpont et al. (62)	García-Sánchez et al. (30)	Delpont et al. (12)	Correia-Gomes et al. (67)	Horie et al. (36)	Tilli et al. (9)	Laconi et al. (13)
*Total external biosecurity*	53.09	58.77	48.11	42.80	52.38	70.28	69.87	62.67	52.97	74.47
Purchase one-day-old chicks	/	/	/	/	/	/	60.53	/	/	70.15
Depopulation broilers	/	/	/	46.00	/	/	46.05	/	/	68.70
Feed and water	39.67	/	50.00	56.50	100.00	/	97.37	/	7.60	84.35
Removal manure and carcasses	/	/	/	48.00	/	/	/	/	53.41	79.42
Visitors and farmworkers	/	58.53	55.68	23.33	/	73.20	59.02	50.00	80.65	68.64
Material supply	/	/	/	50.00	/	/	71.05	/	53.42	/
Infrastructure and biological vectors	66.51	59.00	38.64	37.78	57.15	67.35	75.88	80.00	66.35	71.23
Location farm	/	/	/	38.00	/	/	/	58.00	56.37	/
*Total internal biosecurity*	54.12	71.07	31.82	29.00	25.00	64.40	60.67	66.00	79.55	85.24
Disease management	/	/	31.82	26.00	/	/	63.16	75.00	/	93.30
Cleaning and disinfection	62.78	71.07	/	32.00	25.00	64.40	63.60	57.00	79.55	77.19
Materials and measures between compartments	45.45		/	/	/	/	55.26	/	/	70.2
Total overall biosecurity	53.60	64.92	39.96	35.90	38.69	67.34	65.27	64.33	66.26	79.85

/, data not available.

### 3.4 Quality assessment results

The quality appraisal was performed only for the 16 articles containing extractable data on biosecurity implementation evaluated through scoring or descriptive methods. The results are presented in Supplementary material 2. Overall, the methodological quality of the studies was deemed good. However, the majority of studies (87.5%) did not provide justification for the chosen sample size.

### 3.5 Synthesis of results

The included studies were grouped according to the type of biosecurity assessment method used, namely descriptive and scoring methods. Table 4 shows the pooled levels of biosecurity implementation (percentage of farmers implementing a biosecurity measure) based on descriptive data from 1,692 farms. In general, 58% of farms implemented all the biosecurity measures assessed in the study. External and internal biosecurity measures were implemented by 57 and 60% of farmers, respectively. Measures related to cleaning and disinfection (66.0%, IQR 50.0–89.0%) and purchase of one-day-old chicks (65.3%, IQR 43.6–82.2%) showed the highest level of implementation. Less than half of farmers implemented biosecurity measures related to depopulation (40.2%, IQR 34.5–51.7%) and farm location (45.3%, IQR 30.3–61.3%).

**Table 4:** Level of biosecurity implementation in poultry farms based on descriptive assessments.

Biosecurity measure	Means (IQR)	Number of farms
*External biosecurity*	*57.0*	**–**
Purchase one-day-old chicks	65.3 (43.6–82.2)	106
Depopulation broilers	40.2 (34.5–51.7)	120
Feed and water	64.0 (41.0–97.4)	554
Removal manure and carcasses	61.3 (41.0–86.6)	335
Visitors and farmworkers	60.9 (33.6–87.1)	1,571
Material supply	56.3 (50.0–71.1)	381
Infrastructure and biological vectors	62.9 (47.8–79.2)	1,692
Location of farm	45.3 (30.3–61.3)	365
*Internal biosecurity*	*60.0*	–
Disease management	63.8 (39.7–83.7)	188
Cleaning and disinfection	66.0 (50.0–89.0)	1,670
Materials and measures between compartments	50.4 (47.9–52.8)	227

IQR, interquartile range.

Results based on scoring assessments are presented in Table 5, covering 217 poultry farms. The overall average biosecurity score was 66.9/100. On average, the external and internal biosecurity scores were estimated at 68.0/100 and 65.8/100, respectively. The highest biosecurity scores were recorded for measures related to infrastructure and biological vectors (83.1), and disease management (77.2). Of all the biosecurity subcategories, “feed and water” (48.3) and “material supply” (48.6) received the lowest scores.

**Table 5:** Level of biosecurity implementation in poultry farms based on scoring assessments.

Biosecurity measure	Means ± SD	Number of farms
*External biosecurity*	*68.0 ± 5.6*	*217*
Purchase one-day-old chicks	68.4 ± 3.3	41
Depopulation broilers	61.8 ± 5.3	41
Feed and water	48.3 ± 6.2	41
Removal manure and carcasses	68.4 ± 7.2	41
Visitors and farmworkers	75.2 ± 7.6	41
Material supply	48.6 ± 4.6	41
Infrastructure and biological vectors	83.1 ± 5.2	41
Location farm	66.3 ± 9.6	41
*Internal biosecurity*	*65.8 ± 9.5*	*217*
Disease management	77.2 ± 7.6	41
Cleaning and disinfection	62.2 ± 8.6	41
Materials and measures between compartments	63.8 ± 16.7	41
*Overall*	*66.9 ± 7.5*	

SD, standard deviation.

## 4 Discussion

This systematic review provides an overview of the level of biosecurity implementation in poultry farms across Europe and neighboring regions. Despite continuous research efforts over the past two decades (2003–2023), our findings reveal considerable variability in the implementation of biosecurity practices. This heterogeneity, driven by a wide range of study designs, poultry species, and methodological approaches, highlights the complexity of evaluating biosecurity across diverse national contexts.

This review also highlights the variability in methods used to assess biosecurity, namely descriptive analyses (e.g., reporting the percentage or the number of farms implementing specific measures), scoring systems (e.g., self-scores, Biocheck. Ugent™, national scoring systems, FAO’s Zone Biosecurity model) and probabilistic/simulation models. Similar results have recently been reported by Duarte et al. (25), who identified 33 different methods used to assess biosecurity in poultry farms in Europe and beyond. Descriptive approaches were the most common among the articles analyzed in our study. While such methods provide useful baseline data, the absence of standardized lists and definitions of biosecurity measures poses a challenge for generating comparable outputs across countries (26). In contrast, 13 studies employed scoring systems. Among these, Biocheck. UGent™^5^ was the most commonly used tool, enabling standardized and reproducible assessments of farm biosecurity (27). Probabilistic models (used in 10 studies) also offer valuable insights into the likelihood of pathogen introduction and spread under specific farm conditions (28). However, these models often rely on simulated data rather than empirical on-farm assessments (29). The integration of scoring systems with probabilistic modeling could serve as a standardized and powerful tool for national and regional surveillance, benchmarking, and supporting farm-level decision-making. By combining both systems, a comprehensive approach to data collection and analysis could help identify emerging trends and high-risk areas, thereby reinforcing national and regional surveillance programs. At the farm level, predictive models based on real-world data can support evidence-based decisions, such as optimizing vaccination timing against diseases or intensifying biosecurity measures during high-risk periods (e.g., for avian influenza introduction from wild birds).

Based on descriptive data from 1,692 farms, 58% of farms implemented all the biosecurity measures assessed. In addition, external and internal biosecurity measures were implemented by 57 and 60% of farmers, respectively. However, these values may not fully reflect the actual level of biosecurity implementation, as many studies using descriptive methods assessed the presence of a measure rather than its correct application. For example, in García-Sánchez et al. (30), the question “Does the farm have a vehicle wheel disinfection system?” did not assess whether the system was actually used. Future studies using descriptive methods should include tools that verify whether the measure, when present, is also implemented correctly (31). From the scoring evaluation, the overall average biosecurity score was 66.9/100 with external and internal biosecurity scoring 68.0/100 and 65.8/100, respectively. The majority of these studies focused on broiler farms, highlighting the efforts of EU countries over the last decade to strengthen biosecurity in poultry production. Regulatory frameworks, including Regulation (EU) 2016/429 and related national policies (32), have further supported implementation. Additionally, this likely reflects the economic relevance and industrial standardization of broiler production (33), but also the ease of data collection linked to their shorter production cycles.

Notable differences emerged across specific biosecurity subcategories. Measures related to cleaning and disinfection and the purchase of one-day-old chicks were most commonly implemented in studies using descriptive methods. In contrast, infrastructure and biological vectors, and disease management scored highest in studies using scoring systems. This divergence may be linked to differences in evaluation principles: descriptive evaluations do not assign weights, whereas scoring systems apply risk-based weightings (34).

Most studies (34 out of 44) were pathogen- or disease-specific (e.g., *Campylobacter* spp., *Salmonella* spp., and avian influenza), while only 10 studies focused on evaluating biosecurity measures. This suggests that biosecurity is still perceived as a reactive intervention within epidemiological frameworks rather than a proactive management strategy (30, 35, 36). A shift toward a more holistic and integrative approach, capable of preventing multiple hazards, would foster more resilient poultry health systems.

A majority of studies (77%) focused on a single poultry species, primarily broilers (55%), while assessments involving layers or turkeys were far less frequent. Although this review included solely primary research studies, this imbalance reveals a gap in biosecurity assessments for other systems (e.g., extensive or organic poultry farming, turkeys, and minor poultry species), which may also pose potential zoonotic risks (37).

Some studies (23%) evaluated multiple poultry categories, offering broader insights but often without disaggregated results by production type. Almost all such studies (7 out 10) were conducted during disease outbreaks (especially avian influenza) involving multiple poultry types in a region. For example, Knight-Jones et al. (38) administered a questionnaire during an avian influenza outbreak to holdings within 10 km of infected premises, regardless of species. In Sweden, biosecurity data were collected during a major avian influenza outbreak affecting over 2.2 million birds (39). While these studies offer comprehensive overviews, they often lack production-type–specific detail. Given that avian influenza affects various poultry species, future outbreak-based studies should enable comparisons between broilers, layers, and turkeys. Ssematimba et al. (40), for example, conducted detailed interviews with 42 farmers and 18 poultry business representatives during the H7N7 epidemic in the Netherlands. Their sample was adjusted to represent the national poultry population, allowing insights into biosecurity variation across production types. Future research should therefore combine species-specific and cross-cutting approaches when evaluating biosecurity protocols.

Despite broad geographical coverage—including eight multi-country studies with 36 national assessments—the distribution of studies remains uneven. Countries such as the United Kingdom, France, and the Netherlands are well represented, while others (e.g., Cyprus, Finland, Greece, Ireland, Kosovo, Serbia, Sweden, Tunisia, and Turkey) are covered by only a single study. This imbalance may reflect differences in research funding, national priorities, or logistical barriers to conducting longitudinal studies, or differences in the impact of national poultry production at European level.

Most reviewed studies targeted conventional systems (73%), while few addressed organic or free-range production systems. Given the growing demand for products from systems perceived to offer higher welfare (41), future studies should investigate biosecurity in these contexts. Expanding research into non-conventional systems would help fill critical knowledge gaps and support policy development.

### 4.1 Limitations

This systematic review has some limitations. First, only published original research articles were included, limiting the pool of available studies; in some countries, biosecurity data may exist but not publicly accessible. In some cases, country-specific data were missing, and despite attempts to contact corresponding authors, no further information was obtained, leading to the exclusion of those studies. Second, methodological heterogeneity such as differences in assessment tools/methods, species, definitions, and study designs posed challenges in synthesizing and interpreting the data. These factors may limit the generalizability of the findings and underscore the need for harmonized research protocols in future biosecurity assessments.

## 5 Conclusion

This study aimed to systematically review the level of biosecurity implementation in poultry farms across Europe and neighboring countries. The findings indicate that biosecurity implementation is highly variable, with notable differences in both geographical coverage and the types of poultry systems assessed. Several key areas for improvement emerged from this review, including: (i) the need for more published data on layers, turkeys, and other poultry types beyond broilers, to develop a more comprehensive understanding of biosecurity across diverse systems; (ii) the promotion of cross-country collaboration, capacity building, and targeted resource allocation to enhance research output, data harmonization, and knowledge sharing; and (iii) increased research on alternative (e.g., extensive and organic) poultry production systems, to better understand how production models influence biosecurity implementation and effectiveness.

Addressing these gaps will strengthen future efforts to implement and monitor biosecurity measures, ultimately reducing disease transmission risks and supporting a safer, more resilient poultry sector.

## Data Availability

The original contributions presented in the study are included in the article/Supplementary material, further inquiries can be directed to the corresponding author.

## Footnotes

^1^ https://better-biosecurity.eu/

^2^ https://hdl.handle.net/11577/3511729

^3^ https://www.syreaf.org/protocol/

^4^ https://www.rayyan.ai/

^5^ https://biocheckgent.com/en

## Supplementary material

Supplementary material 1 Search strategy performed in CAB Abstract.

Supplementary material 2 Results of the quality appraisal of included studies.
