# Supplementary material for: Biosecurity implementation in poultry farms across Europe and neighboring countries: a systematic review
Source: Front Vet Sci. 2025 Sep 19;12:1653543. doi: 10.3389/fvets.2025.1653543 (PMC12491017; doi:10.3389/fvets.2025.1653543)
Supplement: Supplementary material 1 — Search strategy performed in CAB Abstract. [file Data_Sheet_1.ZIP › Supplementary material 2.docx]

**Supplementary material 2.** Results of the quality appraisal of included studies with Quantitative Methods-AXIS Checklist.

|  | | | **DA1** | **DA2** | **DA3** | **DA4** | **DA5** | **DA6** | **DA7** | **DA8** | **DA9** | **DA10** | **DA11** | **DA12** | **DA13** | **DA14** | **DA15** | **DA16** |
| --- | --- | --- | --- | --- | --- | --- | --- | --- | --- | --- | --- | --- | --- | --- | --- | --- | --- | --- |
| **Introduction** | 1 | Were the aims/objectives of the study clear? | Yes | Yes | Yes | Yes | Yes | Yes | Yes | Yes | Yes | Yes | Yes | Yes | Yes | Yes | Yes | Yes |
| **Methods** | 2 | Was the study design appropriate for the stated aim(s)? | Yes | Yes | Yes | Yes | Yes | Yes | Yes | Yes | Yes | Yes | Yes | Yes | Yes | Yes | Yes | Yes |
|  | 3 | Was the sample size justified? | No | No | No | No | No | No | Yes | Yes | No | No | No | No | No | No | No | No |
|  | 4 | Was the target/reference population clearly defined? (Is it clear who the research was about?) | Yes | Yes | Yes | No | Yes | Yes | Yes | Yes | Yes | Yes | Yes | Yes | Yes | No | No | Yes |
|  | 5 | Was the sample frame taken from an appropriate population base so that it closely represented the target/reference population under investigation? | Yes | No | Yes | Do not know/ comment | Yes | Do not know/ comment | Yes | No | Do not know/ comment | Yes | Do not know/ comment | No | No | No | No | No |
|  | 6 | Was the selection process likely to select subjects/participants that were representative of the target/reference population under investigation? | Yes | No | Yes | Do not know/ comment | Yes | Do not know/ comment | Yes | No | Do not know/ comment | Yes | Do not know/ comment | No | No | No | No | Yes |
|  | 7 | Were measures undertaken to address and categorise non-responders? | Yes | Do not know/ comment | Do not know/ comment | Do not know/ comment | Do not know/ comment | Do not know/ comment | Yes | Do not know/ comment | | Yes | Do not know/ comment | Do not know/ comment | No | No | No | Yes |
|  | 8 | Were the risk factor and outcome variables measured appropriate to the aims of the study? | Yes | Yes | Yes | Yes | Yes | Yes | No | Yes | Yes | Yes | Yes | Yes | Yes | Yes | No | Yes |
|  | 9 | Were the risk factor and outcome variables measured correctly using instruments/measurements that had been trialed, piloted or published previously? | No | No | Yes | Yes | Yes | Yes | No | No | Yes | Yes | Yes | Yes | Yes | Yes | No | Yes |
|  | 10 | Is it clear what was used to determined statistical significance and/or precision estimates? (eg, p values, CIs) | Yes | Yes | Yes | No | Yes | Yes | Yes | Yes | Yes | Yes | Yes | Yes | Yes | Yes | No |  |
|  | 11 | Were the methods (including statistical methods) sufficiently described to enable them to be repeated? | Yes | Yes | Yes | No | Yes | Yes | Yes | Yes | No | Yes | Yes | Yes | Yes | Yes | No | Yes |
| **Results** | 12 | Were the basic data adequately described? | Yes | Yes | Yes | Yes | Yes | Yes | Yes | Yes | Yes | Yes | Yes | Yes | Yes | Yes | Yes | Yes |
|  | 13 | Does the response rate raise concerns about non-response bias? | No | No | No | No | No | No | Yes | No | No | Yes | No | No | No | No | No | No |
|  | 14 | If appropriate, was information about non-responders described? | Yes | No | No | Do not know/ comment | No | Yes | Yes | Do not know/ comment | | Yes | No | Yes | Do not know/ comment | Yes | Do not know/ comment | Yes |
|  | 15 | Were the results internally consistent? | Do not know/ comment | Do not know/ comment | Do not know/ comment | Yes | Do not know/ comment | Do not know/ comment | Do not know/ comment | Do not know/ comment | Do not know/ comment | Do not know/ comment | Do not know/ comment | Do not know/ comment | Do not know/ comment | Do not know/ comment | Do not know/ comment | Do not know/ comment |
|  | 16 | Were the results for the analyses described in the methods, presented? | Yes | Yes | Yes | Yes | Yes | Yes | Yes | Yes | Yes | Yes | Yes | Yes | Yes | Yes | Yes | Yes |
| **Discussion** | 17 | Were the authors’ discussions and conclusions justified by the results? | Yes | Yes | Yes | Yes | Yes | Yes | Yes | Yes | Yes | Yes | Yes | Yes | Yes | Yes | No | Yes |
|  | 18 | Were the limitations of the study discussed? | Yes | Yes | No | No | No | No | Yes | Yes | No | No | Yes | No | Yes | No | No | Yes |
| **Other** | 19 | Were there any funding sources or conflicts of interest that may affect the authors’ interpretation of the results? | No | No | No | No | No | No | No | No | No | No | No | No | No | No | No | Do not know/ comment |
|  | 20 | Was ethical approval or consent of participants attained? | Yes | Yes | Do not know/ comment | Do not know/ comment | Do not know/ comment |  | Do not know/ comment | Yes | Yes | Do not know/ comment | Do not know/ comment | Yes | Yes | Do not know/ comment | Do not know/ comment | Do not know/ comment |

DA1: Delpont et al., 2018; DA2: Laconi et al., 2023; DA3: Sylejmani et al., 2016; DA4: Sylejmani et al., 2016 ; DA5: García-Sánchez et al., 2020; DA6: Delpont et al., 2021; DA7: Correia-Gomes et al., 2021; DA8: Horie et al., 2021; DA9: Tilli et al., 2022; DA10: Gosling et al., 2014; DA11: Luiken et al., 2019; DA12: Delpont et al., 2019; DA13: Caekebeke et al., 2020; DA14: Schreuder et al., 2023; DA15: Fraser et al., 2010; DA16: Gelaude et al., 2014.
