# Supplementary material for: Biosecurity implementation in poultry farms across Europe and neighboring countries: a systematic review
Source: Front Vet Sci. 2025 Sep 19;12:1653543. doi: 10.3389/fvets.2025.1653543 (PMC12491017; doi:10.3389/fvets.2025.1653543)
Supplement: Supplementary material 1 — Search strategy performed in CAB Abstract. [file Data_Sheet_1.ZIP › Supplementary material 1.docx]

**Supplementary material 1.** Search strategy performed in CAB Abstract.

1 ("chicken*" or "poultry*" or "gallus" or "broiler*" or "layer*" or "turkey" or "meleagri*" or "duck*" or "anas" or "breeders*" or "geese" or "goose" or "fowl*" or "avian*" or "bird*" or "hen" or "hens" or "flock*").mp. [mp=abstract, title, original title, broad terms, heading words, identifiers, cabicodes] 817140

2 ("biosecurity" or "farm biosecurity" or "animal biosecurity" or "preventive veterinary medicine" or "flock health management").mp. [mp=abstract, title, original title, broad terms, heading words, identifiers, cabicodes] 8442

3 ("assess*" or "level*" or "implement*" or "measure*" or "scor*" or "questionnaire*" or "checklist*" or "practice*" or "compliance" or "adopt*").mp. [mp=abstract, title, original title, broad terms, heading words, identifiers, cabicodes] 4382699

4 ("europe*" or "EU" or "Austria*" or "Belgium" or "Bulgaria*" or "Croatia*" or "Cypr*" or "Czech*" or "Denmark" or "Estonia" or "Finland" or "France" or "German*" or "Gree*" or "Hungar*" or "Ireland" or "Ital*" or "Kosovo" or "Latvia" or "Lithuania*" or "Luxembourg" or "Malt*" or "Montenegro" or "Netherlands" or "Macedonia*" or "Norway" or "Poland" or "Portug*" or "Romania*" or "Serbia*" or "Slovakia" or "Slovenia*" or "Spain" or "Sweden" or Belarus or Moldova or "Bosnia and Herzegovina" or "Ukraine" or Andorra or Liechtenstein or Monaco or "Switzerland" or "United Kingdom" or "Tunisi*" or "Turkey" or "Türkiye").mp. [mp=abstract, title, original title, broad terms, heading words, identifiers, cabicodes] 2652590

5 1 and 2 and 3 and 4 526

<https://ovidsp.ovid.com/ovidweb.cgi?T=JS&NEWS=N&PAGE=main&SHAREDSEARCHID=mwj7gTJcqA55b4opElPWp6D08nVfPlEz8F6uknoQ0H50zRbwmuOXQZFSPoaIAwrZ>
